# Practice Gap in Addressing Secondary Tricuspid Regurgitation During Systemic Valve Surgery for Rheumatic Heart Disease: A Retrospective Cohort Study

**DOI:** 10.7759/cureus.91440

**Published:** 2025-09-01

**Authors:** Siddharth V Som, Ankus S Kotwal, Amit Rastogi, Ankit Kumar Sahu, Prabhakar Mishra, Surendra Agarwal, Shantanu Pande

**Affiliations:** 1 Cardiovascular Surgery, Sanjay Gandhi Postgraduate Institute of Medical Sciences, Lucknow, IND; 2 Anaesthesiology, Sanjay Gandhi Postgraduate Institute of Medical Sciences, Lucknow, IND; 3 Cardiology, Sanjay Gandhi Postgraduate Institute of Medical Sciences, Lucknow, IND; 4 Bioinformatics, Sanjay Gandhi Postgraduate Institute of Medical Sciences, Lucknow, IND; 5 Cardiovascular and Thoracic Surgery, Sanjay Gandhi Postgraduate Institute of Medical Sciences, Lucknow, IND

**Keywords:** long term followup, outcome analysis, rheumatic valvular heart disease, secondary tricuspid regurgitation, valve replacement surgery

## Abstract

Background & aims: This study analyses the practice gap in addressing tricuspid regurgitation (TR) in patients undergoing left-sided valve surgery.

Methods: This is a retrospective cohort study of patients operated on between January 2015 and December 2018. A total of 1,129 patients underwent left-sided valve operations for rheumatic aetiology. Tricuspid valve repair (TVr) was performed in 68 patients. Patients with follow-up data and isolated or predominantly mitral valve replacement were divided based on the severity of TR: Group 1 (n = 771), patients with mild TR; Group 2 (n = 109), patients with severe TR and severe pulmonary arterial hypertension (PAH); Group 3 (n = 33), patients with severe TR and non-severe PAH; and Group 4 (n = 68), patients with moderate to severe TR and a dilated tricuspid valve annulus. Group 4 patients received TVr. The primary endpoint was the appearance of severe TR at follow-up.

Results: The mean age of the whole cohort of 1,129 patients (n = 598 (53%) males and n = 531 (47%) females) was 35.42 ± 13.91 years. Mean follow-up of 27.3 ± 18.9 months was available for 1,061 patients. The primary endpoint was observed in 6.3%, 21.1%, 49.5%, and 23.5% of patients, respectively, from Group 1 to Group 4. Preoperatively, mild TR was observed in 771, moderate in 147, and severe in 143 patients who completed follow-up. The primary endpoint was more common in patients with moderate TR (p = 0.04). The American Heart Association (AHA) and American College of Cardiology (ACC) collaborate to produce clinical practice guidelines providing evidence-based recommendations to improve cardiovascular health. These guidelines are the official policy of both organisations and are intended to provide a foundation for quality cardiovascular care globally, though the focus is on US medical practice. There was a departure from the recommendation in 66.6% of cases (Groups 2 and 3) involving patients with severe TR (Groups 2-4). The emergence of severe TR in Groups 2 and 4 was similar, even though patients in Group 2 did not receive TVr, contrary to the recommendation.

Conclusion: The appearance of severe TR during follow-up for patients with preoperatively severe TR with severe pulmonary hypertension (PH) was similar, irrespective of whether TVr was performed. This group should be further investigated regarding the need for TVr.

## Introduction

Tricuspid valve regurgitation has been a fairly common occurrence in patients with left-sided heart valve disease [1]. The estimated incidence is 8%-35%, and it has been considered functional in aetiology [2]. However, organic tricuspid valve disease has also been reported, mostly in patients presenting with chronic rheumatic heart disease [3]. It was long believed that tricuspid regurgitation (TR) would regress following correction of the lesion in the left-sided cardiac valves (the original idea) [4,5]. A study by Nath et al. in 2004 followed patients with TR for four years and revealed that not only did the regurgitation increase, but it was also associated with increased mortality [6]. The results of a large registry also found that patients with uncorrected severe TR at the time of operation for left-sided heart valve disease had higher mortality [7]. These findings led to the inclusion of severe TR as a Class I indication in the American Heart Association/American College of Cardiology (AHA/ACC) and European guidelines [8,9]. Factors influencing surgeons’ decisions to differ from these recommendations include small body surface area, young age, and absence of dilatation of the pulmonary annulus in patients with severe pulmonary arterial hypertension (PAH) and severe tricuspid valve regurgitation undergoing surgery for left-sided valve lesions [10]. This study was conducted to observe adherence to the recommendations by surgeons for tricuspid valve repair (TVr) at the time of surgery for left-sided cardiac valve lesions, and its effect on TR during follow-up.

## Materials and methods

This is a retrospective cohort study conducted at a single institution, involving multiple surgeons from the department. The study included patients operated on between January 2015 and December 2018. It was approved by the Institutional Ethics Committee and was granted a waiver of written consent from the patients included. During the study period, a total of 1,129 patients were included for data analysis, of which 661 underwent mitral valve replacement, 248 had combined aortic and mitral valve replacement, and 182 had aortic valve replacement. Concomitant valve replacement and atrial septal defect closure were performed in 11 patients, and coronary artery bypass grafting in 27 patients, respectively. A total of 981 operations were performed for rheumatic heart valve disease, with preoperative information available on the grade of TR; these patients were included for further analysis. Data were collected from the hospital information system, an electronic database used for archiving patient records. Operative data were reviewed to determine whether a procedure was performed on the tricuspid valve, and if so, what type of procedure was done. Patients with sufficient data were finally divided into four groups based on the severity of TR, indexed tricuspid annulus size, and PAH, along with the requirement for tricuspid annuloplasty as per AHA/ACC guidelines: Group 1 (n = 771), patients with mild TR (including patients with no TR, trivial TR, or mild TR, with or without severe PAH, who did not require tricuspid annuloplasty); Group 2 (n = 109), patients with severe TR and severe PAH (with no annular dilatation, but requiring tricuspid annuloplasty); Group 3 (n = 33), patients with severe TR and non-severe PAH (with dilated tricuspid annulus warranting annuloplasty); and Group 4 (n = 68), patients with moderate to severe TR and dilated tricuspid annulus (requiring tricuspid annuloplasty due to annular dilatation). Two-dimensional echocardiography reports were used to collect data on left ventricular size and function, and TR - preoperatively, one month postoperatively, and annually thereafter, until five years of follow-up were completed. Echocardiography data on TR were available for 981 patients, and complete echocardiographic data were available preoperatively for 824 patients, and for 238 patients at three-year follow-up. Patients with missing data in the hospital information system were contacted by telephone or searched in outpatient visit records. Data were censored in the absence of any postoperative information. Loss to follow-up was attributed to a change of address, relocation, socioeconomic factors, or death.

Patients undergoing left-sided heart valve operation, aged over 18 years and scheduled for elective surgery, were included in the study, while those requiring reoperation or emergency surgery were excluded. During follow-up, the appearance of severe TR was considered the primary endpoint of the study. The event mentioned further in the article refers to the appearance of severe TR in the cohort during follow-up.

Assessment of TR

TR was assessed by 2D echocardiography and Doppler (GE Healthcare, Chicago, IL, USA) in the apical four-chamber view. The assessment was based on the percentage regurgitant jet area compared to the right atrial area. A value of <25% was labelled as mild, 26%-49% as moderate, and >50% as severe [9]. The primary endpoint of the study was the appearance of severe TR.

Surgical procedure

All patients in this study were operated on through a median sternotomy, after general anaesthesia and aseptic preparation and draping. Cardiopulmonary bypass was instituted using bi-caval cannulation for venous drainage and ascending aortic cannulation for return, after full heparinisation, achieved by administering 4 mg/kg body weight of heparin. Left-sided cardiac valve replacement was performed through the left atrium for the mitral valve, and through the aorta for the aortic valve, after aortic cross-clamping and intermittent antegrade delivery of St. Thomas cardioplegia under mild hypothermia. The tricuspid valve procedure was performed through the right atrium after snaring of the vena caval cannulae. The procedures performed were either a modified De Vega annuloplasty or annuloplasty using Teflon felt [11].

Decision-making for TVr

Rheumatic pathology was the aetiology in all the patients in this study. The decision-making in these patients was based on AHA/ACC guideline recommendations [8]. Groups 1 and 4 followed the recommendation, while Groups 2 and 3 did not adhere to the recommendations, based on reasons detailed further in this section. All the patients who presented with severe TR with severe PAH were considered for repair. Further decision-making was based on the diameter of the annulus of the tricuspid valve. If annular dilatation (indexed tricuspid valve annulus diameter of >21 mm/m²) was present, they were considered for repair [12]. However, the patients with severe TR and severe PAH, without dilation of the tricuspid valve annulus, were not repaired. The patients with severe TR without severe PAH received TVr. Moderate TR was addressed with TVr if the annulus was dilated above the cutoff value, as discussed above. Mild TR was left unoperated. However, the operating surgeon’s decision at the time of operation was final and was influenced by factors like younger age, functional tricuspid valve regurgitation, smaller mitral valve area, severe PAH, and absence of atrial fibrillation. These factors, in combination, have been reported to predict regression of TR after mitral valve procedure, by Hannoush et al. [10]. Moreover, the presence of right ventricular dysfunction was considered a reason to avoid TVr in some cases, even when it was recommended [13]. This may have created a bias in the repair of TR.

The decision to avoid TVr in Group 3 was made by the surgeon, who considered it high-risk for the patient due to the presence of - either individually or in combination - right ventricular dysfunction, poor nutritional status, and a higher NYHA (New York Heart Association) classification of heart failure based on symptoms during physical activity. All the patients requiring TVr received modified De Vega’s annuloplasty.

During follow-up, assessment by echocardiography was performed after one month and then annually. The maximum follow-up duration in this study was five years.

Statistical analysis

The variables have been represented as mean and standard deviation or median (interquartile range). Comparison of means between paired groups was performed using a paired t-test. The p-value was considered significant if it was <0.05. Measurement of the agreement between the surgeon’s choice for repair and the recommendation for the patient - expressed as kappa statistics and proportions - was compared by a two-proportion Z test. Survival analysis was performed using Kaplan-Meier curves with the log-rank test, and graphs were prepared using Stata Statistical Software, Version 15 (StataCorp LLC, College Station, TX, USA). Other statistical analyses were performed using IBM SPSS Statistics for Windows, Version 23 (Released 2015; IBM Corp., Armonk, NY, USA).

## Results

The mean age of patients was 35.42 ± 13.91 years, of whom 531 (47%) were females and 598 (53%) were males, with a body surface area of 1.60 ± 0.29 (Table 1). The details of the operations performed are described in Table 2. The patients had a mean follow-up of 27.3 ± 18.9 months, with a maximum follow-up of 60 months.

**Table 1:** Baseline Characteristics of Cohort (N = 1129) BMI: Body Mass Index; BSA: Body Surface Area

Variables	Mean ± SD/Number (%)
Age (years)	
Mean ± SD	35.42 ± 13.91 years
Range	10-76 years
Sex	
Male	598 (53%)
Female	531 (47%)
Height (cm)	154.67 ± 24.17
Weight (kg)	49.00 ± 15.50
BMI	20.97 ± 4.09
BSA	1.60 ± 0.29
Follow-up months	27.3 ± 18.9
Range of follow-up months	0-60 months

**Table 2:** Description of Procedure Done at Primary Operation AVR: Aortic Valve Replacement, CABG: Coronary Artery Bypass Grafting, DVR: Double Valve Replacement, MVR: Mitral Valve Replacement

Type of operation (n = 1,129)	Number (%)
MVR	661 (58.4)
DVR	248 (22)
AVR	182 (16.2)
Combined valve and CABG	27 (2.4)
Atrial septal defect	11 (1)

**Figure 1:**
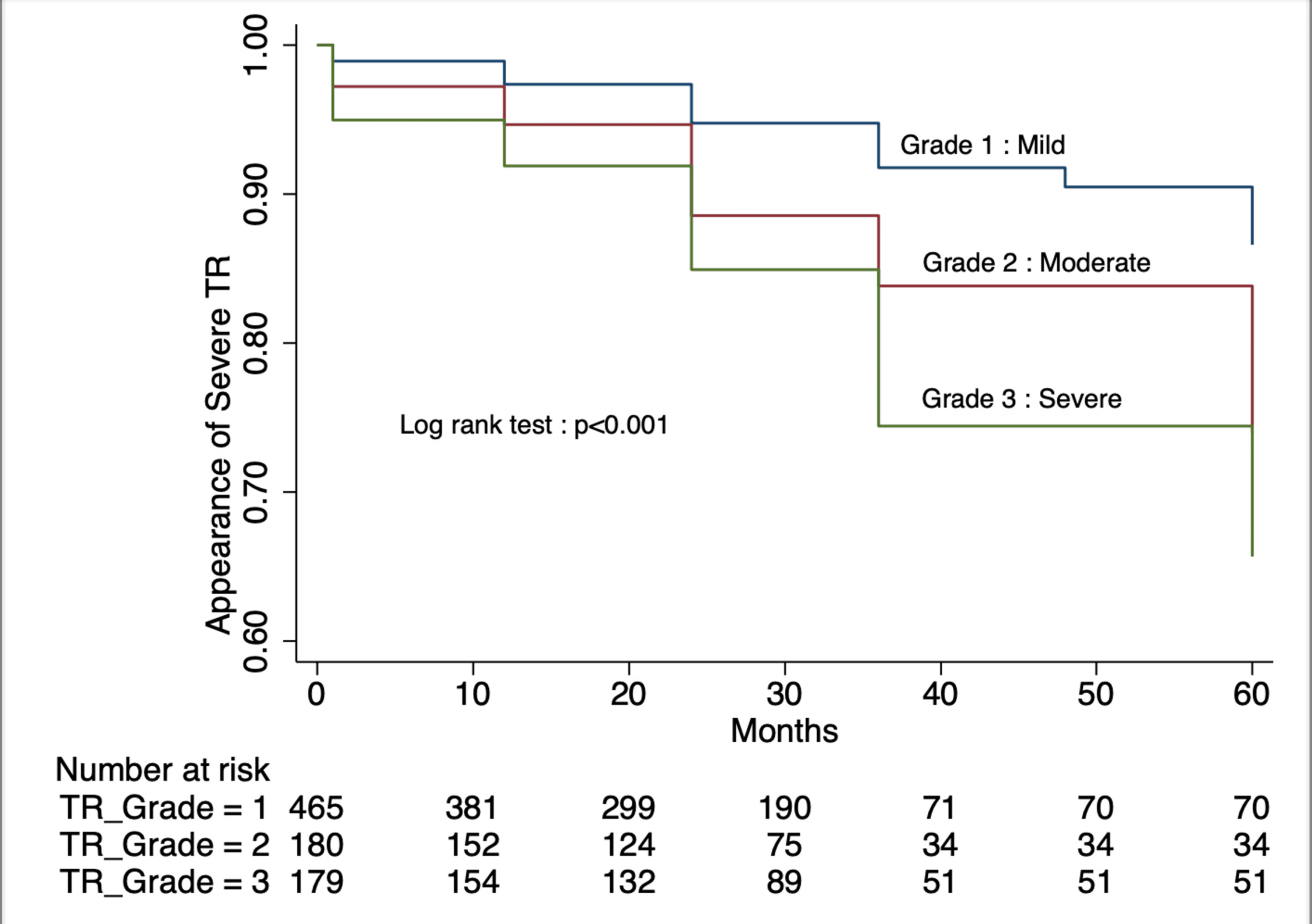
Appearance of Severe TR During Follow-Up Based on Preoperative Status of TR Survival curve representing the appearance of severe tricuspid regurgitation during follow-up in cohorts based on preoperative tricuspid regurgitation status. A p-value of <0.05 was considered significant. TR: Tricuspid Regurgitation

There was improvement in left ventricular function, as witnessed by a statistically significant improvement in left ventricular size and reduction in right ventricular systolic pressure at three years of follow-up (Table 2). The preoperative TR status is shown in Figure 1. During follow-up, there was the appearance of severe TR in all groups, but it was most common in the preoperative moderate TR group.

Figure 1 reveals the appearance of severe TR in different severities of TR preoperatively during follow-up, independent of any surgical approach to address the tricuspid valve. The distribution of severe TR during follow-up in all four groups is presented in Table 3.

**Table 3:** Function of the Left Ventricle Preoperatively and at Five-Year Follow-Up Data presented in mean + standard deviation, compared using a paired t-test. A p-value of <0.05 was considered statistically significant. LVEDD: Left Ventricular End Diastolic Dimension, LVESD: Left Ventricular End Systolic Dimension, LVEF: Left Ventricular Ejection Fraction, RVSP: Right Ventricular Systolic Pressure

Variable	Preoperative value (n = 238)	Three-year follow-up value (n = 238)	T-value	p-value
LVESD in mm	30.84 + 9.51	29.34 + 7.56	3.61	<0.001
LVEDD in mm	49.59 + 10.75	44.95 + 7.58	9.48	<0.001
LVEF in %	59.79 + 4.23	59.18 + 4.11	1.22	0.65
RVSP in mm Hg	55.88 + 21.81	34.84 + 14.85	13.93	<0.001

Most events of severe TR during follow-up were witnessed in the group with the absence of severe PAH but with severe TR, where repair of the tricuspid valve was recommended but not attempted by the operating surgeon. However, there was no difference in the appearance of severe TR in patients who had preoperative severe TR with severe PAH and tricuspid valve left unrepaired versus patients with severe TR who received TVr by modified De Vega’s annuloplasty.

**Figure 2:**
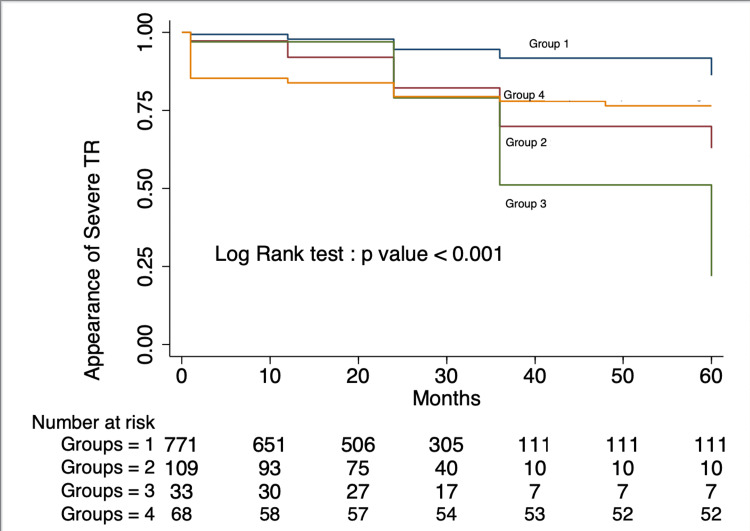
Appearance of Severe TR Based on Study Groups Survival curve representing the appearance of severe tricuspid regurgitation during follow-up, based on study groups. A p-value of <0.05 was considered significant. TR: Tricuspid Regurgitation

The follow-up event-free survival is presented in Figure 2, which displays the appearance of severe TR during follow-up in patients with a combination of different preoperative grades of TR, PAH, and tricuspid procedure.

There was agreement with the recommendation for repair of the tricuspid valve between operating surgeons and the AHA/ACC recommendation when the whole cohort was evaluated. The kappa value was 0.593, with a p-value of <0.001. However, in cases with severe preoperative TR (Groups 2-4), which require TVr as per the recommendation, there was disagreement with the recommendation in 66.6% of cases (Groups 2 and 3). Severe TR appearance during follow-up for each group is presented in Table 4

**Table 4:** Appearance of Severe TR During Follow-Up in Each Group (N = 981) Data presented in numbers (%), compared by the two proportions Z test. ACC/AHA: American College of Cardiology/American Heart Association

Groups with recommendations as per AHA/ACC guidelines	Annuloplasty status in the study cohort	Total number	Appearance of severe TR during follow-up	Z-value	p-value
Group 1: Tricuspid annuloplasty not required	Not done	771	49 (6.3%)	5.29	Group 1 vs. Group 2, p < 0.001
Group 2: Tricuspid annuloplasty recommended	Not done	109	23 (21.1%)	3.19	Group 2 vs. Group 3, p = 0.001
Group 3: Tricuspid annuloplasty recommended	Not done	33	16 (49.5%)	2.63	Group 3 vs. Group 4, p = 0.008
Group 4: Tricuspid annuloplasty recommended	Done	68	16 (23.5%)	-	-
Overall		981	104 (10.6%)	-	-

## Discussion

This study included patients with rheumatic heart valve disease involving the mitral, aortic, or both left-sided valves. There were some important differences in the profile of the patients in this study as compared to other studies. These patients were young (in the third decade of their life), and thus, the exposure to severe PAH was for a shorter duration.

The other important observation was a smaller body surface area, and thus, the indexed tricuspid valve annular diameter was better suited to consider tricuspid annular dilatation rather than an absolute value of the annulus. Furthermore, the dilatation of the tricuspid valve annulus was absent in the majority of patients with severe TR. These factors cause difficulty in following the recommendations for the repair of the tricuspid valve [9].

In this cohort of patients, the application of recommendations for tricuspid valve regurgitation was evaluated. It was observed that there was overall agreement with the recommendation on TVr in the study. This was due to the fact that most of the patients had mild TR (465) as compared to those with moderate or severe regurgitation (180 and 179 patients, respectively).

The incidence of severe TR in this study was 10.60% on follow-up, which was lower compared to the incidence reported by Dreyfus et al., of 34% at five years. This rather large difference is due to the factors specific to the cohort already discussed. Another factor is that the relatively low population of rheumatic pathology in their study was only 11% [14]. In rheumatic pathology, the incidence of severe tricuspid valve regurgitation after a mitral valve procedure has been reported to be as high as 68%, but at a longer follow-up [15,16].

The decision for repair in this study was based on a combination of the size of the tricuspid valve annulus, the severity of TR grade, and the presence of PAH. Moderate regurgitation was addressed by repair in 34 patients, and similarly, 34 patients with severe TR received repair. The repair in these cases was based on the presence of a dilated tricuspid annulus on perioperative transoesophageal echocardiography.

However, patients with severe TR and non-severe PAH, along with either right ventricular dysfunction or absence of details on tricuspid valve diameter, were not repaired [17]. This decision was against the recommendation, which states that any severe tricuspid valve regurgitation of secondary origin should be repaired during the operation.

This decision is defended on the grounds that younger age at operation, smaller mitral valve area, and functional TR may lead to regression of the TR during follow-up [18]. Moreover, the tricuspid annulus was not dilated beyond the values mentioned in the recommendation in most of the patients.

The incidence of severe TR during follow-up was similar in Group 2 (severe TR in the presence of severe PAH with unrepaired tricuspid valve) when compared with Group 4 (when the tricuspid valve was repaired). This observation vindicates the decision to avoid repair of the tricuspid valve in Group 2. However, more importantly, it highlights the failure of the De Vega repair for TR.

The observation in this study, about comparable events of severe TR in repaired and unrepaired tricuspid valves, has also been supported by McCarthy et al. They have reported immediate post-procedure failure of tricuspid repair in 14% of patients, irrespective of the strategy of repair [19].

The presence of right ventricular dysfunction has been identified as a factor that may lead to non-regression or reappearance of severe TR during follow-up [20]. This has been observed by our group in previous studies as well. There was an appearance of right ventricular dysfunction even in the absence of pulmonary hypertension (PH), and it was attributed to the aetiology of rheumatic heart disease per se [21,22].

In this study, it was observed that, preoperatively, the group of patients with moderate TR had more events of severe tricuspid valve regurgitation during follow-up when compared to patients with severe or mild tricuspid valve regurgitation. A similar observation was reported by Kaul et al. They studied patients with rheumatic aetiology and right ventricular dysfunction, which was expected to be the possible reason behind their observation [23].

Apart from rheumatic heart disease, advancing age with PAH was also a contributing factor to right ventricular dysfunction. The theory that decreasing back pressure would lead to a reduction in tricuspid valve regurgitation - and thus reduce the presence of severe TR during follow-up - was partially supported in this study, as the primary endpoint was similar in the repaired and unrepaired tricuspid valve groups [24].

However, the part of this theory suggesting that replacement of the mitral valve reduces back pressure - leading to a reduction in pulmonary arterial pressure and left atrial size - has been validated by our group in the past [25].

When repair was avoided by the surgeon during the operation for some reason - although it was recommended - there were the highest incidences of the primary endpoint (appearance of severe TR during follow-up). This was the group with severe tricuspid valve regurgitation and non-severe PAH. The reason ascribed to this practice was the presence of either right ventricular dysfunction or poor nutritional status, along with NYHA Class III or IV symptoms, which warranted a shorter cross-clamp and operating time. This subset, however, had close to a 50% incidence of TR during follow-up. 

TVr was performed in 64 patients, and the decision was based on transoesophageal echocardiography. The decision was made on the basis of tricuspid annular dilation in both the moderate and severe tricuspid valve regurgitation groups. The repair was performed using modified De Vega’s annuloplasty, or De Vega’s annuloplasty supported by a Teflon felt. Severe tricuspid valve regurgitation reappeared in 23.5% of patients during follow-up. In the literature, a 30% progression of TR has been observed in patients who received De Vega’s annuloplasty during the primary valve operation [25]. When pledgets were used to reinforce the sutures, the results of the modified De Vega’s procedure were reported to be superior [11]. 

Limitations

This study has several limitations. It is a retrospective analysis conducted over a long period, and hence, it is difficult to understand the decision-making process of the surgeons at the time of operation. There is significant bias due to the surgeon’s preference for the procedure. Complete information on the assessment of the preoperative tricuspid valve and right ventricular function is not available for all patients. The decision for repair was based on transoesophageal echocardiography, which was also not available in every case. Though this was a single-group analysis, it involved multiple surgeons at a single centre. Due to this, a confounding effect may be present in the interpretation of the study outcomes.

## Conclusions

Despite the retrospective nature of the study, it can be concluded that the presence of preoperative severe tricuspid valve regurgitation of secondary origin was associated with a significantly higher appearance of severe TR during the five-year follow-up, when compared to the group with mild preoperative TR. There was a similar rate of occurrence of severe TR during follow-up in patients with severe TR and severe PH without repair of the tricuspid valve, as compared to patients who received TVr. This finding indicates a subset of patients in whom TR regresses after left-sided heart valve surgery, despite preoperative severe TR and severe PH. Thus, the recommendation for repair of the tricuspid valve in patients with severe TR in the presence of severe PH during surgery for left-sided heart valve disease must be further investigated.
